# The Effectiveness of Patient Education on Laparoscopic Surgery Postoperative Outcomes to Determine Whether Direct Coaching Is the Best Approach: Systematic Review of Randomized Controlled Trials

**DOI:** 10.2196/51573

**Published:** 2024-06-27

**Authors:** Bhagvat Maheta, Mouhamad Shehabat, Ramy Khalil, Jimmy Wen, Muhammad Karabala, Priya Manhas, Ashley Niu, Caroline Goswami, Eldo Frezza

**Affiliations:** 1 California Northstate University College of Medicine Elk Grove, CA United States

**Keywords:** patient, education, surgeries, laparoscopic, postoperative, outcomes, systematic review

## Abstract

**Background:**

As of 2022, patient adherence to postoperative guidelines can reduce the risk of complications by up to 52.4% following laparoscopic abdominal surgery. With the availability of various preoperative education interventions (POEIs), understanding which POEI results in improvement in patient outcomes across the procedures is imperative.

**Objective:**

This study aims to determine which POEI could be the most effective on patient outcomes by systematically reviewing all the POEIs reported in the literature.

**Methods:**

In total, 4753 articles investigating various POEIs (eg, videos, presentations, mobile apps, and one-on-one education or coaching) were collected from the PubMed, Embase, and Scopus databases. Inclusion criteria were adult patients undergoing abdominal laparoscopic surgery, randomized controlled trials, and studies that provided postoperative outcomes. Exclusion criteria included studies not published in English and with no outcomes reported. Title and abstract and full-text articles with POEI randomized controlled studies were screened based on the above criteria through a blinded, dual review using Covidence (Veritas Health Innovation). Study quality was assessed through the Cochrane Risk of Bias tool. The included articles were analyzed for educational content, intervention timing, intervention type, and postoperative outcomes appropriate for a particular surgery.

**Results:**

Only 17 studies matched our criteria, with 1831 patients undergoing laparoscopic cholecystectomy, bariatric surgery (gastric bypass and gastric sleeve), and colectomy. In total, 15 studies reported a statistically significant improvement in at least 1 patient postoperative outcome. None of these studies were found to have an overall high risk of bias according to Cochrane standards. In total, 41% (7/17) of the included studies using direct individual education improved outcomes in almost all surgery types, while educational videos had the greatest statistically significant impact for anxiety, nausea, and pain postoperatively (*P<*.01). Direct group education demonstrated significant improvement in weight, BMI, exercise, and depressive symptoms in 33% (2/6) of the laparoscopic gastric bypass studies.

**Conclusions:**

Direct education (individual or group based) positively impacts postoperative laparoscopic surgery outcomes.

**Trial Registration:**

PROSPERO CRD42023438698; https://www.crd.york.ac.uk/prospero/display_record.php?RecordID=438698

## Introduction

### Background

Adherence to postoperative guidelines can impact the risk of complications by up to 52.4% after laparoscopic surgery, as shown by a 2022 prospective study [1]. The enhanced recovery after surgery (ERAS) protocol is a systematic approach to minimize postoperative pain, complications, and duration of hospital stay in patients undergoing surgical procedures [2-4]. The protocol, established by the ERAS Society, a not-for-profit multiprofessional multidisciplinary medical-academic society, aims to determine the optimal approach for delivering care to patients undergoing surgical procedures, with the goal of facilitating quicker postoperative recovery [4]. The ERAS protocol consists of patient education, preemptive analgesia, and other practical procedures to improve patient outcomes [4,5]. The ERAS protocol continues to be implemented in a wide range of surgical fields and has been shown to significantly decrease patient complications from 35.7% to 16.4% in a prospective cohort study in 2016 [6].

As the ERAS protocol demonstrates, patient compliance after laparoscopic abdominal surgery is essential to reducing postoperative complications [7]. Nonadherence to the recommendations set by the surgical team, such as medication consumption or general lifestyle suggestions, can have a significant impact on postoperative recovery and patient complications [1,8]. For instance, studies have documented that poor compliance in patients undergoing gastric banding surgeries results in poorer outcomes, including reduced weight loss postoperatively [9]. Educating patients on their surgical procedure, potential postoperative consequences, and preventive steps to minimize complications has improved patient compliance and reduced hospital stays following laparoscopic surgery [5,10]. These preemptive measures may play a profound role in mitigating the psychological burden of pain, anxiety, and fear during recovery [11].

### Objectives

As the laparoscopic approach in surgical procedures is considered to be newer, the research following its patient education for postoperative care is limited [12]. To adapt to these novel approaches, modernized educational formats that have been shown to improve surgical patient outcomes include verbal, written, multimedia, mobile apps, and one-on-one or group counseling [11,13,14]. As intervention types continue to be explored, there is no gold standard preoperative education intervention (POEI) that has shown consistent improvement in patient outcomes across the procedures. The aim of this study is to systematically review the literature on POEIs to ascertain which POEI is more effective in improving outcomes in patients undergoing laparoscopic abdominal surgery.

## Methods

Our review adhered to the PRISMA (Preferred Reporting Items for Systematic Reviews and Meta-Analyses) statement and EQUATOR (Enhancing the Quality and Transparency of Health Research) guidelines This protocol is registered in the PROSPERO database (CRD42023438698) [15].

### Search Strategy

A systematic search was performed using 3 databases: PubMed, Embase, and Scopus. The search strategy was developed through an iterative process, using the methodology recommended by the Study Center of the German Society of Surgery, and included key terms related to laparoscopic abdominal surgeries and patient education [16]. The full search algorithm was used to identify potential articles in all 3 databases (Multimedia Appendix 1).

### Article Selection

**Figure 1:**
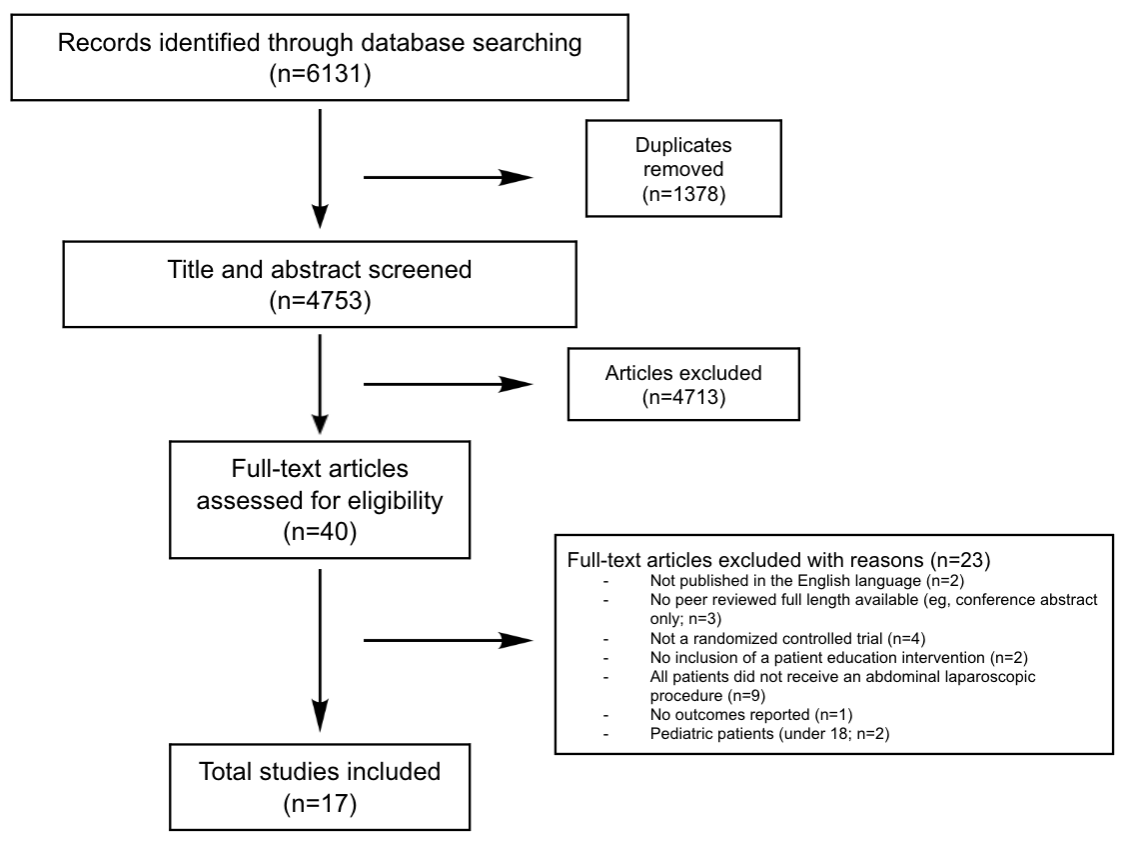
PRISMA (Preferred Reporting Items for Systematic Reviews and Meta-Analyses) flowchart illustrating the process of selecting articles.

A total of 4753 articles investigating POEI were collected from the 3 databases after the removal of duplicates. Inclusion criteria were inclusion of a patient education intervention, adult patients undergoing abdominal laparoscopic surgery, randomized controlled trials (RCTs), and articles including postoperative outcomes (Figure 1). Exclusion criteria were articles not published in English, no patient education intervention included, nonabdominal laparoscopic procedures, pediatric patients, and articles without outcomes reported. Eligibility criteria are described using the population, intervention, comparator, outcomes, timing, and setting framework (Table 1). Title and abstract and full-text articles were screened using the inclusion and exclusion criteria via a blinded, dual review with 2 independent reviewers using Covidence (Veritas Health Innovation). If the decision was not unanimous, discrepancies were resolved after further review until a consensus was reached to determine final article inclusion or exclusion.

**Table 1:** Population, intervention, comparator, outcomes, timing, and setting eligibility criteria.

Domain	Description
Population	Inclusion Adults (ie, aged >18 years) undergoing an abdominal laparoscopic procedure Exclusion Pediatric (ie, aged <18 years) patients Not an abdominal laparoscopic procedure
Intervention	Inclusion Inclusion of a patient education intervention preoperatively including direct individual education (7 studies), direct group education (2 studies), educational video (4 studies), multimedia presentation (2 studies), and mobile app (2 studies). Some education interventions continued postoperatively. Exclusion No inclusion of a patient education intervention
Comparator	Randomized controlled trial Usual preoperative care (eg, surgeon consult and required presurgical routine before bariatric surgery) was the control group. Some interventions included the usual preoperative care along with the education intervention If applicable, preoperative measures were compared to postoperative measures in the intervention group and between intervention and control group
Outcomes	Inclusion Outcomes analyzed Varied between intervention type (ie, nausea, pain, anxiety, fatigue, percentage of unexpected hospitalizations, quality of life, weight, caloric intake, complication rate, first exhaust time, first defecation time, intensive care unit admissions, BMI, exercise, depressive symptoms, Self-Care Mean Agency scores, Body Image Scale scores, and postoperative patient compliance) Exclusion Articles without outcomes reported Outcomes were categorized into 3 categories: patient discomfort, surgical outcomes, and quality of life
Timing	Interventions with any follow-up period were included
Setting	Any care setting (including in-patient clinics or outpatient and ambulatory care)

### Data Extraction and Analysis and Study Quality

Study quality was assessed through the Cochrane Risk of Bias tool as all included studies were RCTs [17]. Each domain assessed (ie, sequence generation, allocation concealment, blinding of participants and personnel, blinding of outcome assessment, incomplete outcome data, selective outcome reporting, and other sources of bias) were evaluated as “high,” “low,” or “unclear” risk of bias. An abstraction form was developed through an iterative process to standardize the data extraction process (Multimedia Appendix 1). Data extraction was performed via a blinded, dual review with 2 independent reviewers on Covidence, with any discrepancies resolved after further review. Study variables analyzed in this systematic review included educational content, intervention timing and duration, intervention type, surgery type, and postoperative outcomes related to a particular surgery. POEIs included educational videos, multimedia presentations, mobile apps, direct individual education, and direct group education. All extracted data were compiled for analysis using Google Sheets (Google Drive; Google, LLC).

## Results

### Literature Selection

Using PubMed, Embase, and Scopus, the initial search yielded 6131 articles, of which 1378 (22.5%) duplicates were removed, leaving 4753 (77.5%) articles. Of the 4753 articles, during the title and abstract screening, we excluded 4713 (99.2%) and included 40 (0.8%). During the second phase, after a full-text review of the 40 articles, 17 (42.5%) were included in this systematic review. From the 17 studies that matched the inclusion criteria, 15 (88.2%) reported a statistically significant improvement in ≥1 patient postoperative outcomes (Table 2) [18-34].

**Table 2:** Summary of the included articles.

Study	Surgery type	Patient demographics	Intervention type (timing+duration)	Content and modality of patient education	Outcome
Abbasnia et al [18]	Laparoscopic cholecystectomy	145 patients (average age 43.54 years) with cholecystitis undergoing laparoscopic cholecystectomy	Educational video (animation 1 shown 2 hours before the surgery and animation 2 shown after the surgery; preoperative and postoperative)	Content Animation 1 was used before surgery to reduce anxiety. “A 40-year-old man entered the operating room with a nurse. History-taking was carried out by an anesthesiologist, and the patient entered the operating room. The equipment and devices that were connected to the patient for monitoring and the method of general anesthesia were shown to the patient. After anesthesia, the recovery room and dressings of the operation site were displayed to the patient. Subsequently, the anatomy of the gall- bladder and its function, as well as the gallbladder surgery by laparoscopy, were demonstrated. Moreover, the patient observed the advantages of the laparoscopy method compared with open surgery.” Animation 2 was used after surgery to manage pain. “A 40-year-old man was seated in a semisitting position, and the narrator states that this condition made it easier to breathe and reduce the pressure inside the abdomen, thereby reducing the pain. Deep breathing and effective coughing were displayed to the patient step by step, and an emphasis was put on the importance of causing faster CO2 (carbon dioxide) gas release from the abdominal cavity and secretions. In addition, the method of fixing the surgical incision with the help of a hand or a small pillow, which helps to reduce pain during coughing, deep breathing, and movement in bed, was demonstrated to the patient. Thereafter, movement in bed was shown to prevent blood clots and encourage faster expulsion of gas from the abdominal cavity. These movements included exercising the sole of the feet, ankles, and thighs. Finally, the patient was shown how to get out of bed step by step.” Modality: virtual reality headsets	There was a statistically significant improvement in preoperative state anxiety, the Bonferroni test for anxiety and patient distraction, pain reported by the VAS^a^, and quality and intensity of subjective pain reported by the McGill Pain Questionnaire.
Bollschweiler et al [20]	Laparoscopic cholecystectomy	76 patients (average age 55.16 years) with cholecystitis undergoing laparoscopic cholecystectomy	Multimedia presentation (preoperative education session was provided)	Content Chapters with disease features, therapeutic alternatives, and the hospital stay, including a description of the operation itself. Certain pages are mandatory for the procurement of informed consent. The chapters focus on the following: Why does the operation need to be performed? The risks of gallstones are presented. Preoperative examinations are described in detail. Complex examinations are presented with videos of each procedure. The chapter explaining that the operative procedure is divided into different sections. The cholecystectomy is clarified using an animated graphic of the operation with a parallel description of the procedure by the surgeon. For interested patients, video from an actual operation is also available. Potential complications from surgery or postoperative risks are related objectively, without focusing on emotional aspects. All risks are shown with rates of occurrence (as described in the literature) and a severity index. Each topic is shown on a navigation bar. By clicking on a risk, background information appears. “The next 4 weeks” chapter includes practical information regarding the length of hospital stay, postoperative nutrition, and aspects of wound treatment for the first 4 weeks after the operation. Modality: in-person with a combination of documents, presentations, and videos	There was a statistically significant improvement in perceived information; however, no statistically significant improvement was found in the Knowledge and Skills Acquisition for anxiety.
da Silva Schulz et al [21]	Laparoscopic cholecystectomy	43 patients (average age 69.35 years) with cholecystitis undergoing laparoscopic cholecystectomy	Direct individual education (ie, fourth, eighth, 12th, 18th, and 25th day postoperative)	Content “The experimental group received the ‘Telephone Consultation’ intervention from a researcher on the 4th (D4), 8th (D8), 12th (D12), 18th (D18) and 25th (D25) postoperative day; a total of 5 telephone consultations were attempted for each participant in the experimental group. During the patient’s follow-up, we used the guidelines developed by NIC standardization and a literature review (e.g., questions about mobility at home, food intake and wound care).” Modality: telephone consultation intervention from a researcher	There was a statistically significant decrease from first to second evaluation and from first to third evaluation for loss of appetite with nausea in the experimental group. Both groups saw a significant decrease from first to third evaluation for pain and reduction was observed in the experimental group for postoperative expectations.
Stergiopoulou et al [30]	Laparoscopic cholecystectomy	60 patients (average age 51.5 years) with cholelithiasis undergoing laparoscopic cholecystectomy	Educational video (20-minute preoperative session was performed in the patient ward; information leaflet and MCD^b^ was available to patients for as long as they wished for)	Content “Multimedia CD contains animation, narration, and photographs with six sections: fundamental elements of bile anatomy and physiology, aspects of the disease, details on the procedure and alternative options, possible complications and duration of hospital stay, and advice about recovery and life after laparoscopic cholecystectomy. Each section has pages, with a total of 28 pages, six of which contained extra photographs and animations. Each page had text fields and the same layout and background graphics. Content was selected in collaboration with surgeons and was written in simple Greek at a senior high school grade level. Leaflet and personalized presentation was developed using the exact contents of MCD.” Modality: multimedia CD with a laptop or leaflet	Groups A, B, and C showed a statistically significant increase in knowledge score regarding laparoscopic cholecystectomy when compared to group D. Furthermore, there was a statistically significant decrease in postoperative pain and nausea during the first 16 hours across all interventional groups when compared to control.
Subirana Magdaleno et al [31]	Laparoscopic cholecystectomy	62 patients (average age 46.8 years) with cholelithiasis undergoing laparoscopic cholecystectomy	Direct individual education (15-30 days before the scheduled surgery; preoperative)	Content Intensified preoperative education with personalized oral and written information of the entire surgical and anesthetic process from a specialized nurse. They were informed about the following points of the process: type of operation, symptoms to be treated in the postoperative period, probable complications, wound care, and diet. Modality: oral and informative brochure	No statistically significant differences were found in terms of pain levels or postoperative nausea, morbidity, percentage of unexpected hospitalizations, quality of life, or degree of satisfaction.
Toğaç and Yılmaz [32]	Laparoscopic cholecystectomy	124 patients (average age 48.72 years) with cholelithiasis undergoing laparoscopic cholecystectomy	Educational video (30- to 45-minute session in 4 stages; preoperative)	Content The first stage included providing information about cholelithiasis, including its causes, preoperative preparation, exercises, surgery, complications, wound care, nutrition, and medicines. Then, a video of laparoscopic cholecystectomy was played on a notebook. Finally, a leaflet about laparoscopic cholecystectomy was shown. In the second stage, knowledge about transfer to the operating room, its physical ambience and waiting room, surgical instruments, and explanations about anesthesia and surgical team were ensured. Information concerning what was expected of the patient before and during general anesthesia and how to join, recovery period, and how the patient is transferred were told. Besides, operating room pictures and surgical instruments were shown via the notebook. In the third stage, photographs and leaflets were used to train patients regarding postoperative care, both in the clinic and at home, such as how to mobilize and change dressing. In the fourth stage, any questions on different issues about laparoscopic cholecystectomy that were not mentioned by the researchers in patient’s education were answered. Afterward, the patients were provided with a leaflet prepared by the researcher to reinforce what they had learned. Modality: photographs, leaflets, and videos	There was a statistically significant decrease in the VAS-pain and VAS-nausea scores of the intervention group at postoperative hours 0, 2, 4, 6, and 8. In addition, the 24-hour VAS-pain score of the intervention group was significantly lower than that of the control group. The VAS-vomiting scores of the control group were higher than those of the intervention group at postoperative hours 6 and 8. Moreover, a significant difference was noted between the intervention and control groups in terms of changes in the VAS-pain, nausea, and vomiting scores over time. Before the intervention, there was no significant difference between the groups in terms of the STAI^c^-I scores; however, a statistically significant difference was determined before surgery and at the postoperative hour 24. There was also a significant difference between the groups in terms of the changes in the STAI-I scores over time. No significant difference was observed between the 2 groups in relation to the STAI-II scores obtained before the intervention, before surgery, and at postoperative hour 24. When the patient learning needs subscale scores were compared before education, there was a significant difference between the 2 groups in terms of activities of living, community and follow-up, feelings related to condition, and enhancing quality of life.
Udayasankar et al [33]	Laparoscopic cholecystectomy	50 patients (average age 40.14 years) undergoing laparoscopic cholecystectomy	Multimedia presentation (preoperative)	Content Information about the surgical procedure and planned anesthetic was given via a PowerPoint presentation on a mobile phone or tablet. The information was a customized collection of graphical representations of surgical and anesthetic procedures that were limited but appropriate. Modality: PowerPoint presentation on a mobile phone or tablet.	Statistically significant reduction was observed in anxiety in ERAS^d^ group compared to control on the day before surgery and 6 hours postoperatively. In addition, there was a statistically significant reduction in hunger, thirst, fatigue, and overall perioperative experience.
Deniz Doğan and Arslan [22]	Laparoscopic gastric bypass	51 patients (average age 38.78 years) undergoing laparoscopic gastric bypass or sleeve gastrectomy	Mobile app (before the operation and first, second, and third months after the operation; preoperative and postoperative)	Content “The app includes care, nutrition, and exercise training for patients undergoing bariatric surgery, starting from the preoperative period, and covering the first 3 months after surgery, as well as a food and an exercise diary, and weight tracking interfaces that will help patients develop healthy lifestyle behaviors while adapting to their new lives. In addition to these, there is a live consultation where patients can communicate with researchers and interfaces with questionnaires and answers to frequently asked questions by patients.” Modality: mobile app and live consultation with researchers and interfaces	There was a statistically significant decrease in the first, second, and third month BMI (kg/m^2^) mean scores of the experimental group; no statistically significant difference was found between Self-Care Mean Agency scores and mean scores of the Body Image Scale.
Kalarchian et al [23]	Laparoscopic gastric bypass	40 patients (average age 46.9 years) undergoing laparoscopic gastric bypass	Direct individual education (4 months of meal plans with monthly individual telephone calls with dietary coach consisting of 4 calls of 15 minute each; postoperative)	Content “That patient intervention included 4 monthly deliveries of portion controlled foods and a personalized menu plan for grocery store items. The participants also received menus that included 3 small meals and 1-2 snacks per day to maintain their portion sizes.” Modality: delivered meal and menu plans	There was a statistically significant improvement in improved weight trajectory and reduced caloric intake relative to a control group.
Kalarchian et al [24]	Laparoscopic gastric bypass	143 patients (average age 44.9 years) with obesity undergoing Roux-en-Y gastric bypass or laparoscopic adjustable gastric banding	Direct individual education (24 weekly contacts, including 12 face- to-face and 12 telephone sessions; postoperative)	Content “consisted of participation in any physician-supervised diet program, in promoting postsurgery weight loss and minimizing complications in comparison with usual care.” Modality: face-to-face and telephone education sessions	There was a statistically significant weight loss from enrollment to postintervention follow-up compared to control. However, at 24 months, the intervention group lost less compared to control.
Mata et al [26]	Laparoscopic gastric bypass	97 patients (average age 59.95 years) undergoing laparoscopic gastric bypass	Mobile app (education intervention was given preoperatively, daily during hospital stay, and at 4 weeks; postoperative)	Content: “Postoperatively, participants randomized to the intervention group received a tablet computer (Apple iPad, Cupertino, USA) containing a novel mobile app. In brief, it included three sections: (1) Milestones checklist: A checklist was always visible in the app’s home page listing the day’s recovery goals with a brief description of the requirements to achieve each one. Next to each description, a button icon was available for the patients to press when the milestone was achieved, and an overall score of the number of milestones achieved compared to the total number for that day was constantly visible in the app’s main dash-board. (2) Daily clinical questionnaires: A brief questionnaire assessing adherence and outcomes for the previous day. In contrast with the milestones checklist, which assessed progress for the present day, the clinical questionnaire assessed the previous day to give an overall summary. Items regarding bowel function and passage of gas were modified for the group of patients with a stoma (i.e., Did you pass stool? Or, did your bag have stool?). After submitting the information, the app displays a total score of the number of ‘milestones met’ (one for every enhanced recovery pathway element of interest they achieved), with a brief phrase of encouragement for goals that were achieved and advice for how to reach the mile-stones that were not yet achieved. Patients could review this feedback at any time in the app’s home page. (3) Education: access to educational material was always available in the app’s home page. Accessing one of the modules produced a detailed description of the milestones for each postoperative day. An exact replica of the education booklet received in their preoperative visit was also included in the educational module.” Modality: novel mobile app on a tablet computer (Apple iPad)	There was no statistically significant improvement of this app on mean adherence to a bundle of 5 postoperative interventions (ie, mobilization, GI motility stimulation, breathing exercises, and consumption of oral liquids and nutritional drinks) that are dependent on patient participation.
Nijamkin et al [28]	Laparoscopic gastric bypass	144 patients (mean age 44.8 years) with obesity undergoing Roux-en-Y gastric bypass surgery.	Direct group education (intervention was given 7 months postoperatively, education was received for 90 minutes every other week for a total of 6 sessions in small groups and frequent contact with a registered dietician; patients were reassessed at 12 months following surgery)	Content “The first session of the education intervention addressed the daily meal planning guide and the maintenance diet. It provided recommendations on identifying and avoiding unhealthful foods, tips to promote proper nutrition by controlling portion size, new routine eating habits, and using an exchange list for weight management. This session was based on the Dietary Guidelines for Americans due to their reliable science-based advice on promoting health and lowering risk for chronic diseases via diet and physical activity. Daily energy intake was limited to 1,000-1,400 kcal and the minimum daily protein intake was 60-70 g with the goal of preserving lean tissue and prevent nutritional deficiencies. Additionally, the session also emphasized characteristics of typical Hispanic diets and the dietary changes that come with acculturation. The session also emphasized traits of typical Hispanic diets and the dietary changes that come with acculturation. Throughout the program, the importance of physical activity and a healthy diet were stressed in the postoperative life. The following session was designed to guide sedentary individuals to begin a regular exercise program and understanding how physical activity can aid in keeping weight off after bariatric surgery. Sessions 3 through 6 focused on emotional support interventions. These include behavior change strategies, stress relief without food, self-motivation, and relapse prevention. Overall, the intervention provided strategies that could facilitate change, increase self-esteem, help establish a consistent exercise program, recognize binge eating problems, and other motivational strategies.” Modality: comprehensive nutrition and lifestyle educational intervention with a registered dietician	At preoperative and 6 months postoperatively, there were no significant differences between intervention and control groups. However, at 12 months, both groups lost significant weight, with the intervention group losing significantly greater weight and significantly greater BMI reduction. Walking mean time, intensity of exercise, and involvement in physical activity was also significantly increased compared to control group at 12 months. No significant difference was found in daily energy intake and number of meals between groups.
Petasne Nijamkin et al [29]	Laparoscopic gastric bypass	144 patients (average age 44.5 years) with obesity undergoing laparoscopic Roux-en-Y gastric bypass	Direct group education (preoperative baseline, 6 months, and 12 months postoperatively)	Content “Those in the comprehensive support intervention received a total of 6 educational sessions focused on behavior change strategies and motivation along with nutrition counseling in groups of up to 12 participants, in addition to the postbariatric standard care. Sessions were conducted every other week in English or Spanish, according to participants’ preference, in a nonjudgmental and nonconfrontational approach, expressing empathy and accepting participants’ unwillingness to change. Group meetings started immediately after the randomization at 6 months after surgery. A psychologist and a registered dietitian guided the educational sessions. Every meeting lasted approximately 90 minutes.” Modality: educational support interventions	Statistically significant decrease of depressive symptoms and greater excess body weight loss were found 12 months after surgery in the interventional group.
Yayla and Menevşe [34]	Laparoscopic sleeve gastrectomy	66 patients (average age 37.09 years) with obesity undergoing laparoscopic sleeve gastrectomy	Educational video (3 times a day at 09 AM, 3 PM, and 9 PM the day before surgery [preoperative] and every postoperative day [days 1-5])	Content “The 9-minute animation education, which was prepared for postoperative sleeve gastrectomy patients, was written and directed by the researchers. The nurse explained how the deep breathing exercise was done using the benefits of respiration exercises (2 minutes) in the first part and the diaphragmatic breathing exercises and incentive spirometry (4 minutes) in the second part. In the third part, the researcher first showed how to do the exercises and then repeated the exercises with the patients (3 minutes).” Modality: animated video sequences	There was a statistically significant difference between the mean postoperative fifth-day pain scores of the experimental and control groups. There was a statistically significant difference between the mean postoperative fifth-day scores of the experimental and control groups.
Li et al [25]	Laparoscopic colectomy	200 patients (average age 55.75 years) undergoing laparoscopic radical resection of colorectal cancer.	Direct individual education (unspecified preoperative or perioperative length, but education continued until discharge)	Content “The preoperative issues were communicated to the patients in ERAS group through face-to-face communication, written notice, or multimedia. Preoperative education includes anesthesia and surgical procedure, encouragement of early postoperative feeding and activity, promotion of pain management and respiratory therapy, presetting discharge criteria, and notification of follow-up and readmission pathway. The education continues through the entire process of the perioperative period until the patient is discharged.” Modality: face-to-face communication, written notice, or multimedia	There were statistically significant differences in complication rate, first exhaust time, and first defecation time between the 2 groups.
Molenaar et al [27]	Laparoscopic colectomy	251 patients (average age 70 years) with colorectal cancer undergoing colorectal cancer resection	Direct individual education (assessments were performed at baseline, preoperatively [approximately 4 weeks after baseline, except for CPET^e^], and 8 weeks postoperatively. Surgical outcomes were evaluated 30 days after surgery)	Content “The supervised training consisted of a 1-hour session of aerobic and strength exercises 3 times per week with resting days in between. The aerobic part, preferably performed on a bicycle, consisted of a high-intensity interval training using baseline CPET-derived variables. It consisted of 4 intervals of 2-minute high-intensity bouts conducted at 85% to 90% of peak power, alternated with 4 intervals of 4-minute moderate intensity bouts at 30% of peak power. Resistance exercise consisted of 2 series of 10 repetitions targeting major muscle groups. The intensity was set at 65% to 70% of the calculated baseline indirect 1 repetition maximum (1 RM). Professional strength equipment, body weight, elastic bands, or calibrated dumbbells were used. Based on nutritional assessment and dietary habits, a registered dietitian provided a full nutritional intervention. The program aimed to balance macronutrients and to achieve a daily amount of proteins of 1.5g per kg. Additionally, participants were provided with a whey protein supplement and were instructed to ingest 30g within 1 hour after the in-hospital training session and 1 hour before sleeping daily. Vitamin D and multivitamin supplements were also provided. Anxiety-coping interventions consisted of relaxation techniques and deep breathing exercises provided by psychology trained personnel in a 1-to-1 session. If a high risk of mental distress was detected by medical history or baseline scores of the Generalized Anxiety Disorder 7-item scale of 10 or higher or Patient Health Questionnaire 9-item of 15 or higher, participants were additionally referred to a medical psychologist. A smoking cessation program was offered, if indicated. The program consisted of individual counseling and nicotine replacement therapy.” Modality: 4-week multimodal personalized in-hospital supervised preoperative program	There was a statistically significant reduction in the rate of severe complications and fewer medical complications observed in patients undergoing prehabilitation compared with standard care. Secondary outcomes regarding admission to intensive care unit were significantly reduced.
Aydal et al [19]	Mixed laparoscopic abdominal surgery	135 patients (average age 43.96 years) undergoing laparoscopic cholecystectomy (n=77, 57%), appendectomy (n=27, 20%), hernia repair (n=15, 11.1%), colon resection (n=7, 5.2%), or gastrectomy (n=6, 4.5%)	Direct individual education (20- to 30-minute preoperative education session)	Content “For the standardization of patient education, an education booklet was prepared in consultation with academic nursing experts. The content included information on the operating room environment and surgical team, anesthesia process, postoperative care, and surgical process. The patient education was not given by the researchers in order to prevent research bias. To avoid any differences between the educators, all education was carried out by one voluntary service nurse and one operating room nurse. About two hours of education was given to the nurses to ensure they adopted a similar approach in patient education and to prevent bias caused by individual factors.” Modality: in-person by a voluntary service nurse and an operating room nurse	There was a statistically significant improvement in anxiety levels (Spielberger State-Trait Anxiety Inventory) directly after the intervention; however, no statistically significant difference was found in anxiety or pain (ie, VAS) levels in the postoperative period.

^a^VAS: Visual Analog Scale.

^b^MCD: multimedia CD.

^c^STAI: State-Trait Anxiety Inventory.

^d^ERAS: enhanced recovery after surgery.

^e^CPET: cardiopulmonary exercise test.

A total of 1831 patients undergoing laparoscopic cholecystectomy, bariatric surgery (ie, gastric bypass and gastric sleeve), and colectomy were included. There were a wide range of patient postoperative outcomes reported in the included studies, including nausea, complication rate, and weight loss (Table 3). These patient outcomes were categorized into *patient discomfort*, *surgical outcomes*, *and quality of life.* No included studies had an overall high risk of bias (Table 4). The PRISMA flowchart illustrates the process of selecting articles in Figure 1 [35].

**Table 3:** Patient education interventions and patient outcomes.

Intervention type (number of studies)	Surgery type	Patient outcomes
Direct individual education (n=7)	Laparoscopic cholecystectomy	Nausea^a^ Pain^a^ Percentage of unexpected hospitalizations Quality of life
Direct individual education (n=7)	Bariatric surgery: laparoscopic gastric bypass	Weight^b^ Caloric intake^a^
Direct individual education (n=7)	Laparoscopic colectomy	Complication rate^a^ First exhaust time^a^ First defecation time^a^ Intensive care unit admission^a^
Educational video (n=4)	Laparoscopic cholecystectomy	Anxiety^b^ Pain^b^ Nausea^a^
Educational video (n=4)	Bariatric surgery: laparoscopic gastric sleeve	Pain^b^
Direct group education (n=2)	Bariatric surgery: laparoscopic gastric bypass	Weight^b^ BMI^b^ Exercise^b^ Depressive symptoms^b^
Multimedia presentation (n=2)	Laparoscopic cholecystectomy	Anxiety^b^ Fatigue^b^
Mobile app (n=2)	Bariatric surgery: laparoscopic gastric bypass	BMI^a^ Self-Care Mean Agency Scores Body Image Scale scores Postoperative patient compliance

^a^*P*<.05.

^b^*P*<.01.

**Table 4:** Risk of bias of the included studies.

Study	Sequence generation	Allocation concealment	Blinding of participants and personnel	Blinding of outcome assessors	Incomplete outcome data	Selective outcome reporting	Other source of bias
Abbasnia et al [18]	Low	Low	Unsure	Unsure	Low	Low	Low
Aydal et al [19]	High	High	High	High	High	Unsure	Low
Bollschweiler et al [20]	Low	Low	High	Low	Low	Low	Low
da Silva Schulz et al [21]	Low	Low	High	Low	High	Low	Low
Deniz Doğan and Arslan [22]	Low	High	High	Low	Low	Low	Low
Kalarchian et al [23]	Low	Low	High	Low	Low	Low	Low
Kalarchian et al [24]	High	High	High	Low	High	Low	Low
Li et al [25]	Unsure	Low	Low	Low	High	Unsure	Low
Mata et al [26]	Low	Low	High	Low	Low	Low	Low
Molenaar et al [27]	Low	Low	High	Low	Low	Low	Low
Nijamkin et al [28]	Low	Low	High	Low	Low	Low	Low
Petasne Nijamkin et al [29]	Low	Low	High	Low	Low	Low	Low
Stergiopoulou et al [30]	High	High	Low	Low	Low	Low	Low
Subirana Magdaleno et al [31]	High	High	High	High	Low	Low	Low
Toğaç and Yılmaz [32]	Low	Low	High	Low	Low	Low	Low
Udayasankar et al [33]	Low	Low	Low	Low	Low	Low	Low
Yayla and Menevşe [34]	Low	Low	High	Low	Low	Low	Low

### Patient Discomfort

The *Patient Discomfort* category consisted of nausea, pain, and anxiety as patient’s postoperative outcomes.

*Nausea* was significantly *(P<*.05) reduced in 2 intervention types. Following laparoscopic cholecystectomy, 43 patients who received direct individual education demonstrated a decrease in postoperative nausea, as measured by the Mini Nutritional Assessment test and the simplified Apfel scale [21]. Educational videos preoperatively also proved to decrease patients’ reporting of nausea [30,32]. The educational video study by Toğaç and Yılmaz [32] was conducted on 124 patients, and the results were obtained using the Visual Analog Scale (VAS). The study by Stergiopoulou et al [30] was conducted on 60 patients, and the results were obtained using the Numerical Rating Scale ranging from 0 to 10. These 2 studies demonstrated statistical significance.

*Pain* was reduced postoperatively following 2 main interventions: direct individual education [21] and educational videos [18,30,32,34]. Direct individual education and educational videos displayed a statistically significant reduction in pain *(P<*.05 and *P*<.01, respectively). The educational video study conducted by Abbasnia et al [18] included 145 patients, and results were obtained with the VAS and McGill Pain Questionnaire. Yayla and Menevşe [34] analyzed 66 patients via the VAS.

*Anxiety* was shown to be statistically decreased *(P<*.01) in POEIs that incorporated both educational videos [18,30] and presentations [33]. The educational video intervention used by Abbasnia et al [18] included 145 patients and collected data via the State-Trait Anxiety Inventory. While Stergiopoulou et al [30] collected data via the Amsterdam Preoperative Anxiety Scale and Information, Udayasankar et al [33] focused on 50 patients and reported a reduction in preoperative anxiety (*P=*.003) and postoperative anxiety after 6 hours (*P=*.001).

### Surgical Outcomes

*Surgical outcomes* category consisted of *percentage of unexpected hospitalizations, complication rate, intensive care unit (ICU) admission, first exhaust time,* and *first defecation time.* These varying patient outcomes provide insight into the patient’s condition after surgery. *Percentage of unexpected hospitalizations* postoperatively was not significantly reduced when direct individual education intervention type was introduced [31]. *Complication rate, ICU admission, first exhaust time,* and *first defecation time* were all reduced postoperatively when patients were debriefed via individual education or coaching intervention [25,27]. Molenaar et al [27] included 251 patients and measured their results via Comprehensive Complication Index (*P*=.02). Li et al [25] obtained their results via observation indicators.

### Quality of Life

Factors that affect *quality of life* were also considered to have a detrimental effect on a patient’s long-term well-being. This category consisted of patient outcome factors such as *weight*, *BMI*, *caloric intake*, *exercise*, *depressive symptoms*, *fatigue*, *Self-Care Mean Agency scores*, and *Body Image Scale scores*. Patient *weight* was found to be statistically significantly decreased in both direct individual and group education POEIs *(P<*.01) [23,24,28,29]. Petasne Nijamkin et al [29] and Nijamkin et al [28] included 144 patients in a group education setting and reported weight loss in patients who received a POEI 12 months postoperatively (*P<*.001). Kalarchian et al [23,24], using a structured intervention, included 40 patients in a direct individual education method and had patients lose weight in the POEI arm at 4 months *(P=*.003).

*BMI* was also found to be statistically significantly decreased in patients provided with direct group education or coaching *(P<*.01) [28] and in patients provided with a POEI using a mobile app *(P<*.05) [22]. Deniz Doğan and Arslan [22] included 51 patients in the mobile app intervention and recorded a reduced BMI *(P<*.05) in the first 3 months postoperatively.

*Caloric intake* was statistically decreased *(P<*.05) when patients received a direct individual education POEI [24]. An increase in *exercise* and a decrease in *depressive* *symptoms* was found to be statistically significant *(P<*.01) when patients received a direct group education POEI [28,29]. In the study by Nijamkin et al [28], exercise was measured via the Short Questionnaire to Assess Health Enhancing Physical. In the study by Petasne Nijamkin et al [29], depression was measured via the Beck Depression Inventory questionnaire and demonstrated a decrease in depression incidence after 12 months *(P<*.001).

Patient *fatigue* postoperatively was decreased when patients were given an educational presentation (*P*=.008) [33]. *Self-Care Mean Agency* scores and *Body Image Scale* scores had no significant increase in patients when provided with a POEI via a mobile app [22]. Deniz Doğan and Arslan [22] assessed *Self-Care Mean Agency scores* via a Likert-type Scale ranging from 0 to 4 with 35 items and *Body Image Scale* via a Likert-type scale ranging from 1 to 5 with 40 items. The direct group education intervention had a significant positive effect on *weight, BMI, exercise,* and *depressive symptoms* for patients after laparoscopic bariatric surgery, suggesting potential future physician consideration as a preferred intervention choice [28,29].

### Direct Individual and Direct Group Education

POEIs included direct individual education, direct group education, video education, multimedia presentations, and mobile apps. Direct individual education methods included supervised and personalized training programs lasting from 1 to 3 months postoperatively as well as nutritional guidance delivered by nurses and physicians via in-person sessions or telehealth [19,27]. POEIs that incorporated personalized training programs led to a decrease in the rate of severe complications (*P<*.05) and anxiety (*P<*.05) [19,27]. Direct individual education also involved personalized preoperative education brochures and advice given by the patient’s surgeon, which reduced nausea postoperatively (*P<*.05) [21]. In addition, patients received postoperative portion-controlled meal deliveries and counseling over 4 weeks, provided by a registered dietitian, leading to weight loss (*P<*.01) and reduced caloric intake (*P<*.05) [24]. Direct group education POEIs for bariatric surgeries involved 4 to 6 comprehensive lifestyle and behavioral or motivational sessions with the research teams and registered dieticians, and it resulted in a significant decrease in weight, BMI, and depressive symptoms (*P<*.01) and a significant increase in exercise (*P<*.01) [28,29].

### Educational Videos and Multimedia Presentations

Video education modalities involved short animations that served the goal of assuaging anxiety and operative fear. These animations were shown to the patient up to 3 times preoperatively and daily postoperatively for 1 week, which led to decreases in anxiety, pain, and nausea (*P<*.01) [18,34]. Likewise, preoperative multimedia presentations administered by registered nurses in the form of CDs and additional animations or brochures provided additional material to the patient before surgery, educating patients about the primary purpose of the surgery, preoperative examinations, and potential complications [20,30,33]. These POEIs led to statistically significant decreases in anxiety and fatigue in patients undergoing laparoscopic cholecystectomy (*P<*.01) [20,30,33].

### Mobile App

Finally, mobile app POEIs developed by the research teams allowed patients to access educational resources on their own time, and it included information about postsurgical care, weight tracking, nutrition, and exercise regimens with recovery goals during the first 3 months of surgery [22,26]. Patients receiving this POEI experienced a decrease in BMI (*P<*.05); however, there was no statistically significant decrease in Self-Care Mean Agency scores, Body Image Scale scores, or postoperative patient compliance [22,26].

## Discussion

### Principal Findings

In this systematic review of RCTs, 17 studies were included, analyzing a total of 1831 patients. Approximately 38% (3/8) of the laparoscopic cholecystectomy studies tested an educational video, which led to a statistically significant decrease in postoperative anxiety, pain, and nausea [18,30,32,34]. Nearly 50% (7/17) of the studies included in this review found that direct individual education improved outcomes for a variety of surgical procedures. Educational videos were most effective at reducing anxiety, nausea, and pain after surgery [18,30]. In about 33% (2/6) of the studies on laparoscopic gastric bypass, direct group education was shown to be effective in improving weight, BMI, exercise, and depressive symptoms. To decrease postsurgery complication rates, ICU admission, as well as first exhaust and defecation time for patients, direct individual education POEIs can be implemented before surgery [25,27].

### Direct Individual Education and Direct Group Education

Direct individual education was the most effective POEI across all included procedure types: laparoscopic cholecystectomy, bariatric surgery, and colectomy [19,21,23-25,27,31]. Direct individual education has been shown to be effective in other surgical procedures as it provides patients with a personalized intervention tailored to their specific needs, which allows for patients to freely communicate and better understand their condition, treatment plan, and postoperative care [36,37]. For example, in hip or knee arthroplasty, patient education led to a significantly shorter length of stay (*P<*.001), suggesting that the effectiveness of one-on-one education or coaching found in this review is not only limited to abdominal laparoscopic procedures [10]. Direct group education had significantly improved outcomes across laparoscopic gastric bypass for weight, BMI, exercise, and depressive symptoms (*P*<.01) [28,29]. A group setting allows for bonding with others and building a support system, which can be a critical influence toward lifestyle changes necessary for improved outcomes after bariatric surgery [38,39]. In a prior systematic review analyzing POEIs in patients undergoing major surgery, the authors found that increased frequency of message exposure improved outcomes; however, this review suggests that the frequency of message exposure may not be as important as POEI type since all frequencies of one-on-one and group education or coaching POEIs had similar effectiveness across all procedure types [13]. Although the included studies incorporated in-person direct individual and group education, there are emerging technologies, such as virtual reality, that offer a new avenue to provide patients with individual or group education and coaching through a distanced modality [40,41].

### Educational Videos and Multimedia Presentations

POEIs with educational videos or a presentation had the most statistically significant improvements on anxiety, pain, and fatigue after laparoscopic cholecystectomy (*P<*.01) [18,20,30,32-34]. The use of videos to educate patients allowed for increased standardization, cost-effectiveness, and accessibility due to the prerecorded nature of this intervention that can be applied broadly throughout multiple disciplines of medicine [42,43]. Incorporation of educational videos also allows for patients to receive the POEI from the convenience of their own home and reduces health care inequity related to access to transportation and proximity to the hospital [44-46]. Preoperative video education has been shown to improve physical symptoms in the literature, as suggested by this review; however, this POEI has also been shown to improve knowledge, preparedness, satisfaction, psychological well-being, quality of life, and health care use in other surgery types [47]. Presentations allow for patients and caregivers to engage with the material and ask questions to better understand the content [48]. Both forms of POEI have demonstrated effectiveness in improving specific patient outcomes based on the content of the education; if the content is tailored toward focusing on additional aspects of the patient’s postoperative recovery, more patient outcomes may be improved [49].

### Mobile Apps

Newer forms of technology are also being tested for POEIs; however, more development is required within this area. In the 2 interventions that leveraged a mobile app for their POEI, there was improvement in BMI (*P<*.01); however, no statistically significant improvement was observed in Self-Care Mean Agency scores, Body Image Scale scores, or postoperative patient compliance [22,26]. Although there were limited significant improvements in patient outcomes while using mobile apps, coupling newer technology with aspects of tested POEIs, such as in-person education, educational videos, or presentations, may be a feasible option to optimize patient outcomes after laparoscopic abdominal surgery. Use of mobile apps in plastic surgery has been shown to significantly improve understanding of the surgery and postoperative patient compliance; this suggests that this modality of POEI has the potential to also improve patient quality outcomes for abdominal laparoscopic procedures if researched further [14]. Benefits of using technology through mobile apps, virtual reality, or artificial intelligence may provide increased accessibility to populations with limited mobility or access to clinical settings. These forms of communication can serve as a vital platform for enhancing the patient-physician rapport [50-53]. There are challenges associated with implementing these tools as the technology of these POEIs encompasses the associated expenses, accessibility, and maintenance. In addition, these platforms will require extensive training to ensure a user-friendly platform for different patient populations [54,55].

### Limitations

This study can be considered in light of the following limitations. First, the tools to report patient outcomes were not consistent across the included studies, thus a meta-analysis or further synthesis is not possible. Second, only laparoscopic cholecystectomy, bariatric surgery (ie, gastric bypass and gastric sleeve), and colectomy surgeries were included because these were the only available surgery types with RCTs published regarding POEI. The heterogeneity of the included studies within the review provides a more diverse and holistic review of the published POEIs, which allows a narrative analysis of the pros and cons of individual interventions in each type of surgery included; however, it limits the ability to statistically compare the interventions to determine the most efficacious POEI in laparoscopic abdominal surgery. There are numerous types of abdominal laparoscopic surgeries where POEI may be beneficial, but they were not included in this systematic review due to a lack of published RCTs. Some included studies did not report all aspects of the POEI, such as information regarding the process of developing the education content or the provision of training, supervision, or assistance with the POEI, including if there was any prototype testing or stakeholder feedback through co-design sessions. This limited the quantification of the effects of these features and their relationship with outcomes as there was significant variability in the published literature. Furthermore, the included studies may have been used for a more comprehensive, multidisciplinary intervention, confounding their direct impact on patient outcomes. However, this study provides informative insights into the current knowledge base pertaining to POEI and its applications in the field of abdominal laparoscopic surgeries.

### Conclusions

This systematic review analyzed 17 RCTs that demonstrated the effect of POEIs on postoperative patient outcomes after abdominal laparoscopic surgeries. A total of 1831 patients undergoing laparoscopic cholecystectomy, bariatric surgery (ie, gastric bypass and gastric sleeve), or colectomy were included in this analysis, and 15 studies reported a statistically significant improvement in at least 1 patient postoperative outcome. Overall, direct individual education was the most effective POEI across all included procedure types; direct group education had the most significantly improved outcomes primarily among bariatric surgeries. POEIs that incorporated educational videos or presentations demonstrated the most statistically significant improvements in anxiety, pain, and fatigue following laparoscopic cholecystectomy. Direct education, whether individual or group based, has been shown to have a more positive impact on postoperative outcomes than newer POEIs, such as mobile apps. The practicality of this allows surgeons to personalize the health care delivered to each patient and provide the appropriate POEI based on which outcomes are more important for that patient. Future directions include expanding the use of POEIs to additional surgical procedures and further testing POEIs that incorporate more recent technology.

## Appendix

Multimedia Appendix 1 Search strategy and abstraction guide.

Multimedia Appendix 2 PRISMA Checklist.

## Abbreviations

EQUATOR: Enhancing the Quality and Transparency of Health Research
ERAS: enhanced recovery after surgery
ICU: intensive care unit
POEI: preoperative education intervention
PRISMA: Preferred Reporting Items for Systematic Reviews and Meta-Analyses
RCT: randomized controlled trial
VAS: Visual Analog Scale
