# Supplementary material for: The Effectiveness of Patient Education on Laparoscopic Surgery Postoperative Outcomes to Determine Whether Direct Coaching Is the Best Approach: Systematic Review of Randomized Controlled Trials
Source: JMIR Perioper Med. 2024 Jun 27;7:e51573. doi: 10.2196/51573 (PMC11240078; doi:10.2196/51573)
Supplement: Multimedia Appendix 1 [file periop_v7i1e51573_app1.docx]

**Appendix S1:** Search Strategy

**PubMed (06/21/2023) - 2,060 results**

("education"[All Fields] OR "patient education"[All Fields] OR "therapeutic education"[All Fields] OR "health education"[All Fields] OR "self-care"[All Fields] OR "self-efficacy"[All Fields] OR "self-management"[All Fields] OR "teaching"[All Fields] OR "empowerment"[All Fields] OR "behavior therapy"[All Fields]) AND ("laparoscopic surgery"[All Fields] OR "laparoscopy"[All Fields] OR "minimally invasive procedure"[All Fields] OR "minimally invasive surgery"[All Fields] OR "bandaid surgery"[All Fields] OR "keyhole surgery"[All Fields] OR "laparoscop*"[All Fields] OR "minimally invasive"[All Fields] OR "microlaparo*"[All Fields] OR "micro laparo*"[All Fields]) AND (("randomized controlled trial"[Publication Type] OR "controlled clinical trial"[Publication Type] OR "random*"[Title/Abstract] OR "placebo"[Title/Abstract] OR "clinical trials as topic"[MeSH Terms] OR "randomly"[Title/Abstract] OR "trial"[Title] OR "clinical trial, phase iii"[Publication Type] OR "phase 3"[Text Word] OR "phase3"[Text Word] OR "phase III"[Text Word] OR "random allocation"[MeSH Terms] OR "double-blind method"[MeSH Terms] OR "single-blind method"[MeSH Terms] OR "clinical trial"[Publication Type] OR "clinical trial, phase i"[Publication Type] OR "clinical trial, phase ii"[Publication Type] OR "clinical trial, phase iv"[Publication Type] OR "multicenter study"[Publication Type] OR "clinical trial*"[Title/Abstract] OR "placebo*"[Title/Abstract] OR "sham"[Title/Abstract] OR "single blind*"[Text Word] OR "double blind*"[Text Word]) NOT (("review"[Publication Type] OR "observational study"[Publication Type] OR "observational study, veterinary"[Publication Type] OR "clinical trial, veterinary"[Publication Type] OR "comparative study"[Publication Type]) NOT "clinical trial"[Publication Type]))

**EMBASE (06/21/2023) - 4,068 results**

('education' OR 'patient education' OR 'therapeutic education' OR 'health education' OR 'self-care' OR 'self-efficacy' OR 'self-management' OR 'teaching' OR 'empowerment' OR 'behavior therapy') AND ('laparoscopic surgery' OR 'laparoscopy' OR 'minimally invasive procedure' OR 'minimally invasive surgery' OR 'bandaid surgery' OR 'keyhole surgery' OR laparoscop* OR 'minimally invasive' OR microlaparo* OR 'micro-laparo*') AND ('randomized controlled trial' OR 'controlled clinical trial' OR 'random*' OR 'placebo' OR 'clinical trials as topic' OR 'randomly' OR 'trial' OR 'clinical trial, phase iii' OR 'phase 3' OR 'phase3' OR 'phase iii' OR 'random allocation' OR 'double-blind method' OR 'single-blind method' OR 'clinical trial' OR 'clinical trial, phase i' OR 'clinical trial, phase ii' OR 'clinical trial, phase iv' OR 'multicenter study' OR 'clinical trial*' OR placebo* OR sham OR 'single blind*' OR 'double blind*')

**Web of Science (06/21/2023) - 1,569 results**

('education' OR 'patient education' OR 'therapeutic education' OR 'health education' OR 'self-care' OR 'self-efficacy' OR 'self-management' OR 'teaching' OR 'empowerment' OR 'behavior therapy') AND ('laparoscopic surgery' OR 'laparoscopy' OR 'minimally invasive procedure' OR 'minimally invasive surgery' OR 'bandaid surgery' OR 'keyhole surgery' OR laparoscop* OR 'minimally invasive' OR microlaparo* OR 'micro-laparo*') AND ('randomized controlled trial' OR 'controlled clinical trial' OR 'random*' OR 'placebo' OR 'clinical trials as topic' OR 'randomly' OR 'trial' OR 'clinical trial, phase iii' OR 'phase 3' OR 'phase3' OR 'phase iii' OR 'random allocation' OR 'double-blind method' OR 'single-blind method' OR 'clinical trial' OR 'clinical trial, phase i' OR 'clinical trial, phase ii' OR 'clinical trial, phase iv' OR 'multicenter study' OR 'clinical trial*' OR placebo* OR sham OR 'single blind*' OR 'double blind*')

**Appendix S2:** Abstraction Guide

**Identification**

**Study Details**

Sponsorship source: *The source of funding for this project.*

Country:

Setting:

Publication date:

Comments:

**Author’s contact details**

Author’s name:

Institution:

Email:

Address:

**Methods**

Design:

**Population**

Inclusion Criteria: *Write out the study’s inclusion criteria for participants*

Exclusion Criteria: *Write out the study’s exclusion criteria for participants*

Group Differences: *If no differences, type “N/A” (because the studies should be randomized controlled trials, there likely will be no significant difference within the groups that impact the analysis of the outcomes).*

**Baseline Characteristics**

| **Characteristics** | **Intervention Arm** | **Control** | **Overall** |
| --- | --- | --- | --- |
| **Sample Size** (N) |  |  |  |
| **Gender** | N male (%)  N female (%)  N other (%) | N male (%)  N female (%)  N other (%) | N/A |
| **Age**  Mean (SD) |  |  | N/A |
| **Race/Ethnicity** | American Indian/Alaska Native: N (%)    Asian: N (%)    Black or African American:  N (%)    Native Hawaiian/Other Pacific Islander: N (%)    Hispanic or Latino: N (%)    White: N (%) | American Indian/Alaska Native:  N (%)    Asian: N (%)    Black or African American:  N (%)    Native Hawaiian/Other Pacific Islander: N (%)    Hispanic or Latino: N (%)    White: N (%) | N/A |
| **Type of illness** | *What type of illness are the participants of the study suffering from to warrant surgical intervention (eg. colon cancer, appendicitis, gall stones, etc…)?* | *What type of illness are the participants of the study suffering from to warrant surgical intervention (eg. colon cancer, appendicitis, gall stones, etc…)?* | *What type of illness are the participants of the study suffering from to warrant surgical intervention (eg. colon cancer, appendicitis, gall stones, etc…)?* |
| **Type of surgical procedure** | *What type of surgery did participants undergo (eg. Laparoscopic Cholecystectomy, etc…)* | *What type of surgery did participants undergo (eg. Laparoscopic Cholecystectomy, etc…)* | *What type of surgery did participants undergo (eg. Laparoscopic Cholecystectomy, etc…)* |

**Interventions**

Overall Directions: *Some boxes will not be applicable to certain studies, and thus can be filled in with “N/A.”*

| **Question** | **Intervention Arm** | **Control** |
| --- | --- | --- |
| **Arm description** | *Copy paste all the details included in the article* | *Copy paste all the details included in the article* |
| **Setting** | *Where is the* ***intervention*** *taking place (eg. in-patient/hospital, clinic based/ambulatory, skilled nursing facilities, home, other)?* | *Where is the* ***control arm receiving care*** *(eg. in-patient/hospital, clinic based/ambulatory, skilled nursing facilities, home, other)?* |
| **Follow up timepoints and duration** | *At what time points were outcomes collected (eg. baseline, 3 months, 6 months, etc…)?* | *At what time points were outcomes collected (eg. baseline, 3 months, 6 months, etc…)?* |
| **Describe the purpose of the patient education intervention** | *What was the purpose of the patient education (eg. educate patients on the symptoms they would expect, motivate them to eat more nutritious food, etc…)?* | *N/A* |
| **Describe the content of the patient education intervention** | *What was covered during the patient education (eg. the importance of eating healthy after surgery, etc…)?* |  |
| **Who was involved in the patient education intervention?** | *Eg. Nurses, physicians, research team members, etc…* |  |
| **What was the role of those involved in the patient education intervention** | *What were the specific responsibilities of each team member involved in the patient education intervention (eg. nurses prepared a PowerPoint presentation, etc…)?* |  |
| **Was there training of team members prior to the intervention?** | *Yes/ No*  *If yes, describe the training provided* |  |
| **Was there technology integration with patient education?** | *Yes/ No*  *If yes, specify what was used and how it was used (eg. zoom was used to communicate with patients, phone calls were used, a TV was used to play an educational video, etc…)* |  |
| **Duration of session, frequency of session, duration of the patient education intervention** | *Eg. 2 hours, twice a day for 3 weeks* |  |
| **Was the patient education intervention used in combination with another treatment?** | *If yes, what treatment?* |  |
| **What were the patient outcomes?** | *List all the patient outcomes* | *List all the patient outcomes* |
| **Did patient outcomes improve?** | *If yes, list which outcomes improved and designate if significant improvement.*  *If not, list the major outcomes that were measured and indicate there was no significant improvement.* | *If yes, list which outcomes improved and designate if significant improvement.*  *If not, list the major outcomes that were measured and indicate there was no significant improvement.* |
| **Was the trial reported with the appropriate registries? If so, which one?** | *Write the registry the trial is reported with and write the registration number.* |  |

**Outcomes**

Overall Directions: Each study will likely have different outcomes and different ways they are reporting the outcomes, thus, individual tables will have to be built for each article (some articles might have multiple usable outcomes and will have multiple tables that we will extract).

- Outcome name: *Include the different types of patient reported outcomes (quality of life, depressive symptoms, anxiety symptoms, etc…).*
- Outcome type: Continuous, Dichotomous, Adverse event
- Reported as: Confidence intervals (mean, CI, N), Standard deviation (mean, SD, N), Standard Error (mean, SE, N)
- Outcome group: *usually not applicable*
- Scale: *enter the name of scale, i.e FACT-G*
- Range: *enter the possible range of results, i.e. 0-10*
- Unit of measurement: *usually not applicable*
- Direction*:* Lower is better or Higher is better
- Data value: Is the change calculated from baseline or endpoint (*usually change is calculated from baseline*)
- Notes: *anything else that helps us better understand the results*

- *Save as an individual outcome. Enter the timepoints of follow-up (i.e. Baseline and 9 months)*
- *Add a new outcome for different types of patient reported outcomes if applicable in the study*

**Appendix S3**: Summary of Included Articles

| **Surgery Type** | **Study** | **Patient Demographics** | **Intervention Type** | **Content and Modality of Patient Education** | **Timing + Duration of Patient Education** | **Outcome** |
| --- | --- | --- | --- | --- | --- | --- |
| **Laparoscopic Cholecystectomy** | Abbasnia et al., 2023 | 145 patients (average age: 43.54) with cholecystitis undergoing laparoscopic cholecystectomy | Educational video | Content:  Animation 1 was used before surgery to reduce anxiety. “A 40-year-old man entered the operating room with a nurse. History-taking was carried out by an anesthesiologist, and the patient entered the operating room. The equipment and devices that were connected to the patient for monitoring and the method of general anesthesia were shown to the patient. After anesthesia, the recovery room and dressings of the operation site were displayed to the patient. Subsequently, the anatomy of the gall- bladder and its function, as well as the gallbladder surgery by laparoscopy, were demonstrated. Moreover, the patient observed the advantages of the laparoscopy method compared with open surgery.”  Animation 2 was used after surgery to manage pain. “A 40-year-old man was seated in a semi-sitting position, and the narrator states that this condition made it easier to breathe and reduce the pressure inside the abdomen, thereby reducing the pain. Deep breathing and effective coughing were displayed to the patient step by step, and an emphasis was put on the importance of causing faster CO2 (carbon dioxide) gas release from the abdominal cavity and secretions. In addition, the method of fixing the surgical incision with the help of a hand or a small pillow, which helps to reduce pain during coughing, deep breathing, and movement in bed, was demonstrated to the patient. Thereafter, movement in bed was shown to prevent blood clots and encourage faster expulsion of gas from the abdominal cavity. These movements included exercising the sole of the feet, ankles, and thighs. Finally, the patient was shown how to get out of bed step by step.”  Modality: Virtual Reality headsets | Animation 1 shown 2 hours before the surgery and Animation 2 shown after the surgery. | **Statistically significant** improvement in pre-operative state anxiety, the Bonferroni test for anxiety and patient distraction, pain reported by the Visual Analogue Scale (VAS), and quality and intensity of subjective pain reported by the McGill Pain Questionnaire (MPQ). |
|  | Bollschweiler et al., 2008 | 76 patients (average age: 55.16) with cholecystitis undergoing laparoscopic cholecystectomy | Presentation | Content:  “Chapters with disease features, therapeutic alternatives, and the hospital stay, including a description of the operation itself. Certain pages are mandatory for the procurement of informed consent.  The chapters focus on the following:  1. Why the operation needs to be performed. The risks of  gallstones are presented.  2. Preoperative examinations are described in detail. Complex examinations are presented with videos of each procedure.  3. The chapter explaining the operative procedure has different subdivisions. The cholecystectomy is clarified using an animated graphic of the operation with a parallel description of the procedure by the surgeon. For interested patients, video from an actual operation is also available.  4. Potential complications from surgery and/or postoperative  risks are related objectively, without focusing on emotional aspects. All risks are shown with rates of occurrence  (as described in the literature) and a severity index. Each topic is shown on a navigation bar. By clicking on a risk  background information appears.  5. “The next 4 weeks” chapter includes practical information regarding the length of hospital stay, postoperative nutrition, and aspects of wound treatment for the first 4 weeks  after the operation”  Modality: In person with a combination of documents, presentations, and videos | The education session was provided before the surgery | **Statistically significant** improvement in perceived information, however, **no statistically significant** improvement in the Knowledge and Skills Acquisition (KASA) for anxiety. |
|  | da Silva Schulz et al., 2020 | 43 patients (average age: 69.35) with cholecystitis undergoing laparoscopic cholecystectomy | One-on-one education/ coaching | Content:  “The experimental group received the “Telephone Consultation” intervention from a researcher on the 4th (D4), 8th (D8), 12th (D12), 18th (D18) and 25th (D25) postoperative day; a total of 5 telephone consultations were attempted for each participant in the experimental group. During the patient’s follow-up, we used the guidelines developed by NIC standardization and a literature review (e.g., questions about mobility at home, food intake and wound care). “  Modality:  “Telephone Consultation” intervention from a researcher | 4th, 8th, 12th, 18th, and 25th day post surgery | **Statistically significant** decrease from 1st to 2nd evaluation and from 1st to 3rd evaluation for loss of appetite with nausea in the experimental group.” Both groups saw a **significant** decrease from 1st to 3rd evaluation for pain and reduction was observed in the experimental group for postoperative expectations). |
|  | Stergiopoulou et al., 2007 | 60 patients (average age: 51.5) with cholelithiasis undergoing laparoscopic cholecystectomy | Educational Video | Content:  “Multimedia CD contains animation, narration, and photographs with six sections: fundamental elements of bile anatomy and physiology, aspects of the disease, details on the procedure and alternative options, possible complications and duration of hospital stay, and advice about recovery and life after LC. Each section has pages, with a total of 28 pages, six of which contained extra photographs and animations. Each page had text fields and the same layout and background graphics. Content was selected in collaboration with surgeons and was written in simple Greek at a senior high school grade level. Leaflet and personalized presentation was developed using the exact contents of MCD.”  Modality: Multimedia CD with laptop or leaflet | Preoperative session was performed in the patient ward that lasted for 20 minutes. Information leaflet and MCD was available to patients for as long as they wished for. | Groups A, B, and C showed a **statistically significant increase** in knowledge score regarding LC when compared to group D. Also a **statistically significant decrease** in postoperative pain and nausea during the first 16 hours across all interventional groups when compared to control. |
|  | Subirana Magdaleno et al., 2018 | 62 patients (average age 46.8) with cholelithiasis undergoing laparoscopic cholecystectomy | One-on-one education/ coaching | Content:  Intensified preoperative education with personalized oral and written information of the entire surgical and anesthetic process from a specialized nurse. They were informed about the following points of the process: type of operation, symptoms to be treated in the postoperative period, probable complications, wound care and diet.  Modality: Oral and informative brochure | The intensified preoperative education occurred 15-30 days before the scheduled surgery. | **No statistically significant** **differences** were found in terms of pain levels or postoperative nausea, morbidity, percentage of unexpected hospitalizations, quality of life or degree of satisfaction. |
|  | Toğaç et al., 2021 | 124 patients (average age: 48.72) with cholelithiasis undergoing laparoscopic cholecystectomy | Educational Video | Content: The first stage included providing information about cholelithiasis, including its causes, preoperative preparation, exercises, surgery, complications, wound care, nutrition, and medicines. Then, a video of laparoscopic cholecystectomy was played on a notebook. Finally, a leaflet about laparoscopic cholecystectomy was shown. In the second stage, knowledge about transfer to the operating room, its physical ambience and waiting room, surgical instruments, and explanations about anesthesia and surgical team were ensured. Information concerning what was expected of the patient before and during general anesthesia and how to join, recovery period and how the patient is transferred were told. Besides, operating room pictures and surgical instruments were shown via the notebook. In the third stage, photographs and leaflets were used to train patients regarding postoperative care, both in the clinic and at home, such as how to mobilize and change dressing. In the fourth stage, any questions on different issues about laparoscopic cholecystectomy that were not mentioned by the  researchers in patient’s education were answered. Afterwards, the patients were provided with a leaflet prepared by the researcher to reinforce what they had learned.”  Modality: Photographs, leaflets, videos | The education session was given in 30-45 minutes in four stages preoperatively. | **Statistically significant decrease** in the VAS-pain and VAS-nausea scores of the intervention group at postoperative hours 0, 2, 4, 6, and 8. In addition, the 24-h VAS-pain score of the intervention group was **significant lower** than that of the control group. The VAS-vomiting scores of the control group were higher than those of the intervention group at postoperative hours 6 and 8. Also, a **significant difference** was noted between the intervention and control groups in terms of changes in the VAS-pain, nausea, and vomiting scores over time . Before the intervention, there was **no significant difference** between the groups in terms of the STAI-I scores; however, a **statistically significant difference** was determined before surgery and at the postoperative hour 24. There was also a **significant difference** between the groups in terms of the changes in the STAI-I scores over time. **No significant difference** was observed between the two groups in relation to the STAI-II scores obtained before the intervention, before surgery , and at postoperative hour 24 . When the PLNS subscale scores were compared before education, there was a **significant difference** between the two groups in terms of activities of lving, community and follow-up , feelings related to condition , and enhancing quality of life |
|  | Udayasankar et al., 2020 | 50 patients (average age: 40.14) undergoing laparoscopic cholecystectomy | Presentation | Content:  Surgical procedure and planned anesthetic was given via a Powerpoint presentation on a mobile phone or tablet. The information was a customized collection of graphical representations of surgical and anesthetic procedures that were limited, but appropriate.  Modality: Powerpoint presentation on mobile phone or tablet. | The education session was provided before the surgery. | **Statistically significant reduction** in anxiety in ERAS group compared to control on the day prior to surgery and 6 h postoperatively. In addition, **statistically significant** in reduced hunger , thirst), fatigue , and overall perioperative experience |
| **Laparoscopic gastric bypass** | Deniz Doğan et al., 2022 | 51 patients (average age: 38.78) undergoing laparoscopic gastric bypass or sleeve gastrectomy | Mobile App | Content:  “The app includes care, nutrition, and exercise training for patients undergoing bariatric surgery, starting from the preoperative period, and covering the first 3 months after surgery, as well as a food and an exercise diary, and weight tracking interfaces that will help patients develop healthy lifestyle behaviors while adapting to their new lives. In addition to these, there is a live consultation where patients can communicate with researchers and interfaces with questionnaires and answers to frequently asked questions by patients.”  Modality:  Mobile App, and live consultation with researchers and interfaces | Prior to the operation, 1st, 2nd, and 3rd months after the operation | **Statistically significant decrease** in the 1st, 2nd, and 3rd month BMI (kg/m2) mean scores of the experimental group  **No statistically significant** difference between self care mean agency scores  **No statistically significant** difference between mean scores of the Body Image Scale |
|  | Kalarchian et al., 2016 | 40 patients (average age: 46.9) undergoing laparoscopic gastric bypass | One-on-one education/ coaching | Content:  “That patient intervention included 4 monthly deliveries of portion controlled foods and a personalized menu plan for grocery store items. The participants also received menus that included 3 small meals and 1-2 snacks per day to maintain their portion sizes.”  Modality:  Delivered meal and menu plans. | 4 months of meal plans with monthly individual telephone calls with dietary coach consisting of 4 calls at 15 minutes each | **Statistically significant** improvement in improved weight trajectory and reduced caloric intake relative to a control group. |
|  | Kalarchian et al., 2016 | 143 patients (average age: 44.9) with obesity undergoing roux-en-y gastric bypass or laparoscopic adjustable gastric banding | One-on-one education/ coaching | Content:  “Consisted of participation in any physician-supervised diet program, in promoting postsurgery weight loss and minimizing complications in comparison with usual care.”  Modality:  “Face-to-face and telephone education sessions.” | “24 weekly contacts, including 12 face to face and 12 telephone sessions.” | **Statistically significant** weight loss from enrollment to post-intervention follow-up compared to control. However, at 24 months, the intervention group lost less compared to control. |
|  | Mata et al., 2020 | 97 patients (average age: 59.95) undergoing laparoscopic gastric bypass | Mobile App | Content:  “Postoperatively, participants randomized to the intervention group received a tablet computer (Apple® iPad, Cupertino, USA) containing a novel mobile app. In brief, it included three sections:  (1) Milestones checklist: A checklist was always visible in the app’s homepage listing the day’s recovery goals with a brief description of the requirements to achieve each one. Next to each description, a button icon was available for the patients to press when the milestone was achieved, and an overall score of the number of  milestones achieved compared to the total number for that day was constantly visible in the app’s main dash-board.  (2) Daily clinical questionnaires: A brief questionnaire assessing adherence and outcomes for the previous day. In contrast with the milestones checklist, which assessed progress for the present day, the clinical questionnaire assessed the previous day to give an overall summary. Items regarding bowel function and passage of gas were modified for the group of patients with a stoma (i.e., Did you pass stool? Or, did your bag have stool?). After submitting the information, the app displays a total score of the number of “milestones met” (one for every ERP element of interest they achieved), with a brief phrase of encouragement for goals that were achieved and advice for how to reach the mile-  stones that were not yet achieved. Patients could review this feedback at any time in the app’s home page.  (3) Education: access to educational material was always available in the app’s home page. Accessing one of the modules produced a detailed description of the milestones for each postoperative day. An exact replica of the education booklet received in their preoperative visit was also included in the educational module.”  Modality: Novel mobile app on a tablet computer (Apple iPad) | Patient education intervention was given preoperatively, daily during hospital stay, and 4 weeks following surgery. | There was **no statistically significant** improvement of this app on mean adherence to a bundle of five postoperative interventions (mobilization, GI motility stimulation, breathing exercises, and consumption of oral liquids and nutritional drinks) that are dependent on patient participation. |
|  | Petasne Nijamkin et al., 2012 | 144 patients (mean age: 44.8) with obesity undergoing Roux-en-Y gastric bypass surgery. | Group education/ coaching | Content:  “The first session of the education intervention addressed the daily meal planning guide and the maintenance diet. It provided recommendations on identifying and avoiding unhealthful foods, tips to promote proper nutrition by controlling portion size, new routine eating habits, and using an exchange list for weight management. This session was based on the Dietary Guidelines for Americans due to their reliable science-based advice on promoting health and lowering risk for chronic diseases via diet and physical activity. Daily energy intake was limited to 1,000-1,400 kcal and the minimum daily protein intake was 60-70 g with the goal of preserving lean tissue and prevent nutritional deficiencies. Additionally, the session also emphasized characteristics of typical Hispanic diets and the dietary changes that come with acculturation. The session also emphasized traits of typical Hispanic diets and the dietary changes that come with acculturation. Throughout the program, the importance of physical activity and a healthy diet were stressed in the postoperative life. The following session was designed to guide sedentary individuals to begin a regular exercise program and understanding how physical activity can aid in keeping weight off after bariatric surgery. Sessions 3 through 6 focused on emotional support interventions. These include behavior change strategies, stress relief without  food, self-motivation, and relapse prevention. Overall, the intervention provided strategies that could facilitate change, increase self-esteem, help establish a consistent exercise program, recognize binge eating problems, and other motivational strategies.”  Modality: Comprehensive nutrition and lifestyle educational intervention with a registered dietician. | Patient intervention was given 7 months following the RYGB, education was received for 90 minutes every other week for a total of 6 sessions in small groups and frequent contact with a registered dietician. Patients were reassessed at 12 months following surgery. | At preoperative and 6 months postoperatively, there were **no significant differences** between intervention and control group. However, at 12 months, both groups lost **significant** weight, with the intervention group losing **significantly** greater weight , and **significantly** greater BMI reduction. Walking mean time, intensity of exercise, and involvement in physical activity was also **significantly increased** compared to control group at 12 months. **No significant difference** in daily energy intake and number of meals between groups. |
|  | Petasne Nijamkin et al., 2013 | 144 patients (average age: 44.5) with obesity undergoing laparoscopic Roux-en-Y gastric bypass | Group education/ coaching | Content:  “Those in the comprehensive support intervention received a total of 6 educational sessions focused on behavior change strategies and motivation along with nutrition counseling in groups of up to 12 participants, in addition to the post-bariatric standard care. Sessions were conducted every other week in English or Spanish, according to participants' preference, in a nonjudgmental and non-confrontational approach, expressing empathy and accepting participants' unwillingness to change. Group meetings started immediately after the randomization at 6 months after surgery. A psychologist and a registered dietitian guided the educational sessions. Every meeting lasted approximately 90 minutes.”  Modality: Educational support interventions | Baseline (preoperatively), 6 months following surgery (end of phase 1), and 12 months after surgery (end of phase 2) | **Statistically significant decrease** of depressive symptoms and greater excess body weight (EBW) loss were found 12 months after surgery in the interventional group. |
| **Laparoscopic sleeve gastrectomy** | Yayla et al., 2023 | 66 patients (average age: 37.09) with obesity undergoing laparoscopic sleeve gastrectomy | Educational video | Content:  “The 9-minute animation education, which was prepared for postoperative sleeve gastrectomy patients, was written and directed by the researchers. The nurse explained how the deep breathing exercise was done using the benefits of respiration exercises (2minutes) in the first part and the diaphragmatic breathing exercises and incentive spirometry (4minutes) in the second part. In the third part, the researcher first showed how to do the exercises and then repeated the exercises with the patients (3minutes).”  Modality: Animated video sequences | The animation training was performed with the researcher three times a day at 09:00, 15:00, and 21:00 the day before surgery and every postoperative day (days 1–5). | **Statistically significant difference** between the mean postoperative fifth-day pain scores of the experimental and control groups. There was a **statistically significant difference** between the mean postoperative fifth-day scores of the experimental and control groups. |
| **Laparoscopic Colectomy** | Li et al., 2019 | 200 patients (average age: 55.75) undergoing laparoscopic radical resection of colorectal cancer. | One-on-one education | Content:  “The preoperative issues were communicated to the patients in ERAS group through face-to-face communication, written notice, or multimedia. Preoperative education includes anesthesia and surgical procedure, encouragement of early postoperative feeding and activity, promotion of pain management and respiratory therapy, presetting discharge criteria, and notification of follow-up and readmission pathway. The education continues through the entire process of the perioperative period until the patient is discharged.”  Modality: Face-to-face communication, written notice, or multimedia | Unspecified preoperative/perioperative length, but education continued until discharge | **Statistically significant** differences in complication rate, first exhaust time, and first defecation time between the two groups. |
|  | Molenaar et al., 2023 | 251 patients (average age: 70) with colorectal cancer undergoing colorectal cancer resection | One-on-one education/ coaching | Content:  “The supervised training consisted of a 1-hour session of aerobic and strength exercises 3 times per week with resting  days in between. The aerobic part, preferably performed on a bicycle, consisted of a high-intensity interval training using baseline CPET-derived variables. It consisted of 4 intervals of 2-minute high-intensity bouts conducted at 85% to 90% of peak power, alternated with 4 intervals of 4-minute moderate intensity bouts at 30% of peak power. Resistance exercise consisted of 2 series of 10 repetitions targeting major muscle groups. The intensity was set at 65% to 70% of the calculated baseline indirect 1 repetition maximum (1 RM). Professional strength equipment, body weight, elastic bands, and/or calibrated dumbbells were used. Based on nutritional assessment and dietary habits, a registered dietitian provided a full nutritional intervention. The program aimed to balance macronutrients and to achieve a daily amount of proteins of 1.5 g per kg. Additionally, participants were provided with a whey protein supplement and were instructed to ingest 30g within 1 hour after the in-hospital training session and 1 hour before sleeping daily. Vitamin D and multivitamin supplements were also provided. Anxiety-coping interventions consisted of relaxation techniques and deep breathing exercises provided by psychology trained personnel in a 1-to-1 session. If a high risk of mental distress was detected by medical history and/or baseline scores of the Generalized Anxiety Disorder 7-item scale of 10 or higher or Patient Health Questionnaire 9-item of 15 or higher, participants were additionally referred to a medical psychologist. A smoking cessation program was offered, if indicated. The program consisted of individual counseling and nicotine replacement therapy.”  Modality: 4-week multimodal personalized in-hospital supervised preoperative program. | Assessments were performed at baseline, preoperatively (approximately 4 weeks after baseline) (except for CPET) 8 weeks after surgery. Surgical outcomes, evaluated 30 days after surgery. | **Statistically significant** reduction in rate of severe complications and fewer medical complications observed in patients undergoing prehabilitation compared with standard care. Secondary outcomes regarding admission to ICU were **significantly reduced**. |
| **Mixed laparoscopic abdominal surgery** | Aydal et al., 2022 | 135 patients (average age: 43.96) undergoing laparoscopic cholecystectomy 77 (57%), appendectomy 27 (20%), hernia repair 15 (11.1%), colon resection 7 (5.2%), or gastrectomy 6 (4.5%) | One-on-one education/ coaching | Content:  “For the standardization of patient education, an education booklet was prepared in consultation with academic nursing experts. The content included information on the operating room environment and surgical team, anesthesia process, postoperative care, and surgical process. The patient education was not given by the researchers in order to prevent research bias. To avoid any differences between the educators, all education was carried out by one voluntary service nurse and one operating room nurse. About two hours of education was given to the nurses to ensure they adopted a similar approach in patient education and to prevent bias caused by individual factors.”  Modality: In person by a voluntary service nurse and an operating room nurse | 20-30 minute education session provided before the surgery | **Statistically significant** improvement in anxiety levels (Spielberger State-Trait Anxiety  Inventory) directly after the intervention, however, **no statistically significant** difference in anxiety or pain (Visual Analog Scale) levels in the postoperative period. |

**Appendix S4:** Risk of Bias of Included Studies

| **Study** | **Sequence Generation** | **Allocation Concealment** | **Blinding of Participants and Personnel** | **Blinding of Outcome Assessors** | **Incomplete Outcome Data** | **Selective Outcome Reporting** | **Other Source of Bias** |
| --- | --- | --- | --- | --- | --- | --- | --- |
| Abbasnia et al., 2023 | Low | Low | Unsure | Unsure | Low | Low | Low |
| Aydal et al., 2022 | High | High | High | High | High | Unsure | Low |
| Bollschweiler et al., 2008 | Low | Low | High | Low | Low | Low | Low |
| da Silva Schulz et al., 2020 | Low | Low | High | Low | High | Low | Low |
| Deniz Doğan et al., 2022 | Low | High | High | Low | Low | Low | Low |
| Kalarchian et al., 2016 | Low | Low | High | Low | Low | Low | Low |
| Kalarchian et al., 2016 | High | High | High | Low | High | Low | Low |
| Li et al., 2019 | Unsure | Low | Low | Low | High | Unsure | Low |
| Mata et al., 2020 | Low | Low | High | Low | Low | Low | Low |
| Molenaar et al., 2023 | Low | Low | High | Low | Low | Low | Low |
| Petasne Nijamkin et al., 2012 | Low | Low | High | Low | Low | Low | Low |
| Petasne Nijamkin et al., 2013 | Low | Low | High | Low | Low | Low | Low |
| Stergiopoulou et al., 2007 | High | High | Low | Low | Low | Low | Low |
| Subirana Magdaleno et al., 2018 | High | High | High | High | Low | Low | Low |
| Toğaç et al., 2021 | Low | Low | High | Low | Low | Low | Low |
| Udayasankar et al., 2020 | Low | Low | Low | Low | Low | Low | Low |
| Yayla et al., 2023 | Low | Low | High | Low | Low | Low | Low |
